# Diagnostic performance of various cephalometric parameters for the assessment of vertical growth pattern

**DOI:** 10.1590/2177-6709.21.4.041-049.oar

**Published:** 2016

**Authors:** Maheen Ahmed, Attiya Shaikh, Mubassar Fida

**Affiliations:** 1 Resident in Orthodontics, The Aga Khan University Hospital, Section of Dentistry, Department of Surgery, Karachi, Pakistan.; 2 Consultant Orthodontist/Assistant Professor, Program Coordinator Orthodontics Residency Program, The Aga Khan University Hospital, Section of Dentistry, Department of Surgery, Karachi, Pakistan.; 3 Consultant Orthodontist/Associate Professor, Program Director Orthodontics Residency Program, The Aga Khan University Hospital, Section of Dentistry, Department of Surgery, Karachi, Pakistan.

**Keywords:** Divergence, Cephalometry, Vertical dimension.

## Abstract

**Introduction:**

Multiple cephalometric analyses are used to diagnose vertical skeletal facial discrepancy. A multitude of times, these parameters show conflicting results, and a specific diagnosis is hard to reach.

**Objective:**

Hence, this study aimed to identify the skeletal analysis that performs best for the identification of vertical skeletal pattern in borderline cases.

**Methods:**

The sample consisted of 161 subjects (71 males and 90 females; mean age = 23.6 ± 4.6 years). Y-axis, Sella-Nasion to mandibular plane angle (SN.MP), maxillary plane to mandibular plane angle (MMA), Sella-Nasion to Gonion-Gnathion angle (SN.GoGn), Frankfort to mandibular plane angle (FMA), R-angle and facial height ratio (LAFH.TAFH) were used to evaluate vertical growth pattern on lateral cephalograms. The subjects were divided into three groups (hypodivergent, normodivergent and hyperdivergent groups), as indicated by the diagnostic results of the majority of parameters. Kappa statistics was applied to compare the diagnostic accuracy of various analyses. To further validate the results, sensitivity and positive predictive values (PPV) for each parameter were also calculated.

**Results:**

SN.GoGn showed a substantial interclass agreement (k = 0.850). In the hypodivergent group, MMA showed the highest sensitivity (0.934), whereas FMA showed the highest PPV (0.964). In the normodivergent group, FMA showed the highest sensitivity (0.909) and SN.GoGn had the highest PPV (0.903). SN.GoGn showed the highest sensitivity (0.980) and PPV (0.87) in the hyperdivergent group.

**Conclusions:**

SN.GoGn and FMA were found to be the most reliable indicators, whereas LAFH.TAFH is the least reliable indicator in assessing facial vertical growth pattern. Hence, the cephalometric analyses may be limited to fewer analyses of higher diagnostic performance.

## RESUMO

**Introdução:**

diferentes análises cefalométricas são utilizadas para se diagnosticar a discrepância esquelética vertical da face. Muitas vezes, essas análises revelam resultados conflitantes, tornado complicado obter um diagnóstico preciso.

**Objetivo:**

o objetivo do presente estudo foi identificar a melhor análise esquelética para identificação do padrão esquelético vertical em casos limítrofes.

**Métodos:**

a amostra consistiu em 161 indivíduos (71 homens e 90 mulheres, com idade média de 23,6 ± 4,6 anos). Para avaliar o padrão de crescimento vertical em telerradiografias de perfil, foram utilizados: o eixo Y, o ângulo formado entre a linha sela-násio e o plano mandibular (SN.MP), ângulo formado pelos planos maxilar e mandibular (MMA), ângulo formado pelas linhas sela-násio e gônio-gnátio (SN.GoGn), ângulo formado pelo plano de Frankfort e o plano mandibular (FMA), o ângulo R, e a proporção de altura facial (AFAI.AFAT). Os indivíduos foram divididos em três grupos (hipodivergente, neutro e hiperdivergente), em acordo com os resultados diagnósticos da maioria dessas medidas. A análise estatística Kappa foi empregada para comparar a precisão diagnóstica das diferentes análises. Para uma validação adicional dos resultados, também foram calculados, para cada medida, a sensibilidade e os valores preditivos positivos (VPP).

**Resultados:**

o SN.GoGn apresentou um valor alto de concordância interclasses (k = 0,850). No grupo hipodivergente, o MMA apresentou a maior sensibilidade (0,934), enquanto o FMA apresentou o VPP mais alto (0,964). No grupo neutro, o FMA apresentou a maior sensibilidade (0,909), enquanto o SN.GoGn apresentou o VPP mais alto (0,903). No grupo hiperdivergente, o SN.GoGn apresentou a maior sensibilidade (0,980) e o valor mais alto de VPP (0,87).

**Conclusões:**

constatou-se que, para avaliação do padrão de crescimento facial vertical, o SN.GoGn e o FMA foram os indicadores mais confiáveis, enquanto o AFAI.AFAT foi o indicador menos confiável. Sendo assim, a análise cefalométrica pode se limitar às análises com maior desempenho diagnóstico.

## INTRODUCTION

Facial vertical growth pattern plays a vital role in achieving facial balance.^1^ Variations in vertical growth are common and have certain orthodontic implications. A long or a short face may be due to abnormal hard or soft tissues that form the face. Growth excess in the vertical dimension may result in gingival smile, incompetent lips and a long face.^2^ On the contrary, a deficiency in vertical growth may lead to inadequate display of incisors, overclosure of lips and a short face.^3^ Both facial types are considered unesthetic and are included in the orthodontic problem list. Treatment of such problems is usually carried out by functional jaw orthopedics in growing patients and by orthognathic surgery in adults. The success of a treatment plan in Orthodontics is not only dependent on understanding where growth occurs, but also when it ends.^4^ As the vertical component of growth is the last to end, failure to control it may lead to complex treatment, compromised results and relapse after treatment.^5^ ^,^ ^6^ This mandates a thorough assessment and an accurate diagnostic evaluation of such discrepancies in the vertical facial pattern to ensure treatment success.

Lateral cephalometry has made the assessment of vertical skeletal problems an easy and accurate process. Downs,^7^ in 1948, used Frankfort horizontal (FH) plane as the reference line on lateral cephalograms to assess the mandibular diversion pattern, using Y-axis and the Frankfort mandibular plane angle (FMA). Steiner^8^ postulated Sella-Nasion to mandibular plane angle (SN.MP) to assess vertical growth pattern using the anterior cranial base as the reference plane. Schwartz,^9^ using the palatal plane, proposed the maxillary/mandibular planes angle (MMA) to assess intermaxillary relationship in the vertical dimension. Later, linear parameters, which included Jarabak's ratio and facial height ratio (LAFH.TAFH) were also used to assess the facial vertical growth of an individual.^10^

There are various linear and angular analyses for evaluating vertical skeletal growth of an individual. The commonly used angular analyses include Sella-Nasion to Gonion-Gnathion plane angle (SN.GoGn), Sella-Nasion to Gonion-Menton plane angle (SN.MP), Frankfort to mandibular plane angle (FMA), maxillary/mandibular planes angle (MMA) and Y-axis.^7^ ^,^ ^8^ ^,^ ^9^ ^,^ ^11^ The linear parameters used to determine vertical growth pattern include Jarabak's ratio and facial height ratio (LAFH.TAFH).^10^ A literature review showed that all of the aforementioned parameters have some shortcomings in terms of identifying the landmarks.^12^ ^,^ ^13^ Paranhos et al,^12^ in their study, reported the Y-axis to be inadequate to assess vertical dysplasia, as the position of Gnathion (Gn) varies with sagittal malocclusion. Similarly, FMA was considered to be less reliable, as landmarks forming the FH plane are difficult to identify.^13^ To overcome the aforementioned shortcomings, new cephalometric analyses, such as the R-angle, are still being introduced.^14^

Apart from potential errors in landmark identification, the cephalometric norms established by the previous studies may not serve adequately for other population groups. A survey of the pertinent literature also showed that variation within the norms may occur due to ethnic differences. Shaikh and Alvi,^15^ in their study on a sample of Pakistani population, showed a difference in facial height ratio, as compared to Caucasians. In contrast, another study reported a normal facial height of 50-55 % in Pakistani subjects.^16^ Because of these inherited discrepancies, the norms could only be used as a reference and not as absolute values.

Previously, many studies have reported the correlation between various skeletal analyses,^17^ ^,^ ^18^ but only a few have compared the diagnostic accuracy and the applicability of the various analyses.^19^ Moreover, during cephalometric analysis, certain cases present with a wide range of readings and not all the parameters used to assess vertical growth indicate a specific pattern. Hence, this study aimed to identify the skeletal analysis that performs best for the identification of vertical facial pattern in borderline cases. Thus, unnecessary analysis can be eliminated, leading to an efficient treatment plan. 

## MATERIAL AND METHODS

A cross sectional study was conducted with the data collected from the diagnostic records of patients visiting the dental clinics of the authors. Keeping a power of study (β) as 80%, α = 0.05, and using the correlation value (r) = 0.168, as reported by Asad and Naeem,^18^ sample size was calculated to be 126. This number was inflated by 10%, which showed that we needed at least 135 subjects.

A total of 161 subjects (71 males and 90 females; mean age = 23.6 ± 4.6 years), aged between 18-35 years old, having clear and good quality cephalograms, were included in the study, whereas those with a history of growth problems, trauma or previous orthodontic treatment were excluded.

**Figure 1:**
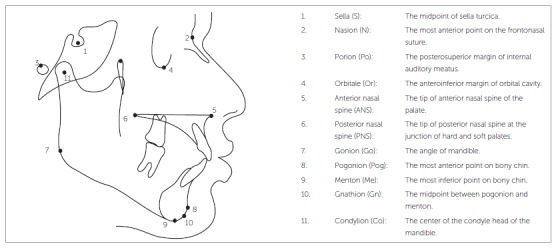
Skeletal landmarks.

Pretreatment lateral cephalograms were used to evaluate vertical skeletal patterns. The distance from the imaging device to the midsagittal plane of the patient was kept constant at 60 cm, and the distance from the film to the midsagittal plane was kept at 15 cm. Cephalograms were traced by hand on matte acetate paper, with a 0.5-mm lead pencil, over an illuminator, by the main investigator using the conventional method. Skeletal landmarks were identified (Fig 1). Measurements were taken with the help of a millimeter ruler and a protractor. 

**Figure 2:**
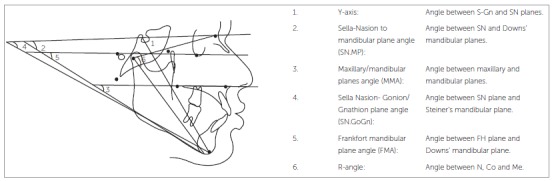
Skeletal angular parameters.

The angular parameters were measured as described bellow (Fig 2):

» Y-axis: The angle between S-Gn and SN planes.
» Sella-Nasion to mandibular plane angle (SN.MP): Angle between SN and Downs' mandibular planes.
» Maxillary/mandibular planes angle (MMA): The angle between maxillary and mandibular planes.
» Sella-Nasion to Gonion-Gnathion angle (SN.GoGn): The angle between SN and Steiner's mandibular planes.
» Frankfort mandibular plane angle (FMA): The angle between FH and Downs' mandibular plane.
» R-angle: The angle between N, Co and Me.

**Figure 3:**
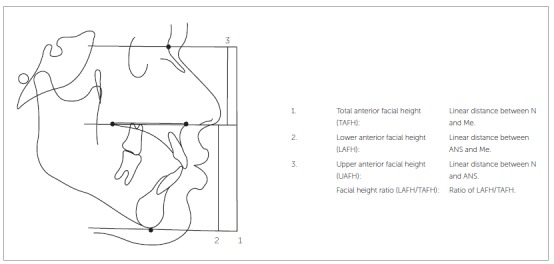
Skeletal linear parameters.

The skeletal linear parameters were the following (Fig 3):

» Total anterior facial height (TAFH): Linear distance between N and M.
» Lower anterior facial height: (LAFH): Linear distance between ANS and Me.
» Facial height ratio (LAFH.TAFH): The ratio of LAFH/TAFH.

The cephalometric norms of each skeletal analysis previously established in the literature were used in the study (Table 1). On the basis of norms of each parameter, the subjects were labeled as hypodivergent, normodivergent and hyperdivergent. Nineteen subjects were shown to have the same facial pattern by all the parameters and thus were eliminated based on a clear-cut diagnosis. Each of the remaining 161 subjects had at least one cephalometric analysis giving conflicting diagnosis of the vertical facial pattern. The final diagnosis of vertical growth pattern of these subjects was based on the results of the majority of parameters, which enabled us to divide our sample into hypodivergent, normodivergent or hyperdivergent. Thus, the division of our sample resulted in the following groups: 

**Table 1:** Cephalometric norms of skeletal analyses.

Parameter	Hypodivergent	Normodivergent	Hyperdivergent
SN.GoGn^8^	< 28°	28°-36°	>36°
FMA^7^	< 21°	21°-29°	>29°
MMA^9^	< 21°	21°-29°	>29°
Y-Axis^8^	<61°	61°-68°	>68°
SN.MP^7^	< 28°	28°-36°	> 36°
Facial height ratio (LAFH.TAFH)^16^	< 50%	50-55 %	> 55%
R-angle^14^	< 70.5°	70.5°-75.5°	> 75.5°

» Low angle: 46.
» Normal angle: 66.
» High angle: 49.

The cases in which diagnosis of a particular cephalometric parameter matched the final diagnosis were regarded as the correctly diagnosed cases. The number of correctly diagnosed cases was used to assess the diagnostic performance of each of the cephalometric parameters.

To assess intraexaminer reliability, 30 radiographs were randomly selected and reanalyzed by the main investigator. The intraclass correlation coefficient denoted that the original and the repeated measurements showed a high correlation (Table 2). 

**Table 2:** Intraclass correlation coefficient.

Measurements	1^st^ reading	2^nd^ reading	ICC
	(n = 30)	(n = 30)	
SN.GoGn	28.90 ± 8.49	29.10 ± 8.29	0.997
FMA	25.17 ± 6.61	25.33 ± 6.33	0.994
MMA	21.63 ± 5.98	21.86 ± 6.02	0.996
Y-axis	66.60 ± 4.56	66.66 ± 4.59	0.995
SN.MP	27.83 ± 7.07	27.86 ± 7.12	0.997
ANS-Me	68.10 ± 6.68	68.36 ± 6.62	0.997
Na-Me	52.51 ± 3.71	52.48 ± 3.90	0.990
R-angle	72.53 ± 4.06	72.36 ± 4.11	0.994

ICC: Intraclass correlation coefficient.

n = 30.

Data were analyzed by means of SPSS for Windows (version 20.0, SPSS Inc. Chicago, USA). Various vertical skeletal parameters were correlated by means of Pearson Correlation. Correlations among various skeletal parameters were also determined separately for males and females. Kappa statistics was applied to assess the level of agreement between the diagnostic interpretation of cephalometric parameters and the final diagnosis made from the majority factor. Positive predictive value (PPV) and the sensitivity of each cephalometric analysis were calculated from the two-by-two tables. A *p*-value of < 0.05 was taken as statistically significant.

## RESULTS

The sample consisted of 161 subjects that included 90 females (mean age = 24.65 ± 4.08 years) and 61 males (mean age = 22.48 ± 4.89 years). The subjects were divided into hypodivergent, normodivergent and hyperdivergent groups. The means and standard deviation of each cephalometric parameter are shown in Table 3. The groups were statistically matched on the basis of chronological age and sagittal relationship.

**Table 3:** Mean value of cephalometric parameters.

Parameter	Hypodivergent n = 46 Means ± SD	Normodivergent n = 66 Means ± SD	Hyperdivergent n = 49 Means ± SD
SN.GoGn	24.04 ± 3.37	31.43 ± 3.74	41.97 ± 4.25
FMA	19.53 ± 3.68	25.98 ± 3.27	33.94 ± 4.649
MMA	16.64 ± 3.31	22.44 ± 3.12	29.31 ± 3.48
Y-Axis	59.33 ± 3.25	62.49 ± 3.54	67.06 ± 5.54
SN.MP	23.75 ± 3.82	29.94 ± 3.28	38.16 ± 4.45
LAFH.TAFH	50.46 ± 2.21	50.97 ± 2.83	52.26 ± 3.37
R-angle	70.56 ± 3.06	74.43 ± 2.92	80.00 ± 3.61

SD: standard deviation.

n = 161.

Pearson Correlation was used to determine the correlation between various skeletal analyses. A strong correlation was present between SN.GoGn and FMA (r = 0.874), SN.GoGn and SN.MP (r = 0.852), and SN.GoGn and MMA (r = 0.811). A moderate positive correlation was present between FMA and R-angle (r = 0.724), FMA and SN-GoMn (r = 0.769), FMA and MMA (r = 0.776) and R-angle and MMA (r = 0.675) (Table 4).

**Table 4:** Correlation among various skeletal analyses to assess vertical growth pattern.

	Y-axis	FMA	SN.GoGn	R-angle	SN.MP	MMA	LAFH.TAFH
Y-axis	1	0.592**	0.595**	0.622**	0.489**	0.475**	-0.033
FMA		1	0.874**	0.724**	0.769**	0.776**	0.208**
SN.GoGn			1	0.776**	0.852**	0.818**	0.222**
R-angle				1	0.678**	0.675**	0.243**
SN.MP					1	0.811**	0.131
MMA						1	0.179*
LAFH.TAFH							1

n = 161; Pearson correlation: weak correlation (± 0.01 < r < ± 0.5); moderate correlation (± 0.5 < r < ± 0.8); strong correlation (± 0.8 < r < ± 1). *p < 0.05; ** *p* < 0.01.

Correlation was also determined for sex separately. A strong positive correlation was found between SN.GoGn and SN.MP for both males (r = 0.898) and females (r = 0.806) (Tables 5 and 6).

**Table 5:** Correlation among various skeletal analyses to assess vertical growth pattern in males.

	Y-axis	FMA	SN.GoGn	R-angle	SN.MP	MMA	LAFH.TAFH
Y-axis	1	0.634**	0.644**	0.670**	0.658**	0.570**	-0.144
FMA		1	0.901**	0.802**	0.862**	0.877**	0.298*
SN.GoGn			1	0.821**	0.898**	0.854**	0.260*
R-angle				1	0.779**	0.720**	0.292*
SN.MP					1	0.875**	0.232
MMA						1	0.217
LAFH.TAFH							1

n = 71; Pearson correlation: weak correlation (± 0.01 < r < ± 0.5); moderate correlation (± 0.5 < r < ± 0.8); strong correlation (± 0.8 < r < ± 1). *p < 0.05; ** *p* < 0.01.

**Table 6:** Correlation among various skeletal analyses to assess vertical growth pattern in females.

	Y-axis	FMA	SN.GoGn	R-angle	SN.MP	MMA	LAFH.TAFH
Y-axis	1	0.585**	0.570**	0.599**	0.376**	0.419**	0.050
FMA		1	0.850**	0.669**	0.657**	0.640**	0.119
SN.GoGn			1	0.744**	0.806**	0.782**	0.190
R-angle				1	0.604**	0.650**	0.187
SN.MP					1	0.736**	0.041
MMA						1	0.148
LAFH.TAFH							1

n = 90; Pearson correlation: weak correlation (± 0.01 < r < ± 0.5); moderate correlation (± 0.5 < r < ± 0.8); strong correlation (± 0.8 < r < ± 1). *p < 0.05; ** *p* < 0.01.

Kappa statistics assessed the agreement among diagnostic criteria of various cephalometric analyses. A substantial agreement was present between SN.GoGn and the final group (k = 0.850, *p* < 0.01) (Table 7). 

**Table 7:** Assessment of agreement among diagnositic criteria of skeletal analyses.

Parameter	Hypodivergent	Normodivergent	Hyperdivergent	n =161	
	n = 46	n = 66	n = 49	Kappa	P-value
SNGoGn	49	62	50	0.850**	0.000
MMA	58	77	26	0.590**	0.000
YAxis	110	42	9	0.152**	0.001
FMA	28	84	49	0.711**	0.000
SNGoMn	54	72	25	0.639**	0.000
LAFH/TAFH	50	87	24	0.046*	0.0401
R-Angle	28	74	59	0.561**	0.000

n = 161; Kappa Statistics.

Positive predictive value (PPV) and sensitivity of each diagnostic parameter were also calculated for each group separately. In the hypodivergent group, MMA showed the highest sensitivity (0.934), whereas FMA showed the highest PPV (0.964). In the normodivergent group, FMA showed the highest sensitivity (0.909) and SN.GoGn had the highest PPV (0.903). SN.GoGn showed the highest sensitivity (0.980) and PPV (0.87) in the hyperdivergent group (Table 8). 

**Table 8:** Assessment of agreement among diagnostic criteria of skeletal analyses.

Parameter	Hypodivergent (n = 46)			Normodivergent (n = 66)			Hyperdivergent (n = 49)		
	Correctly diagnosed cases	Positive predictive value	Sensitivity	Correctly diagnosed cases	Positive predictive value	Sensitivity	Correctly diagnosed cases	Positive predictive value	Sensitivity
SN.GoGn	40	0.816	0.869	56	0.903	0.848	43	0.980	0.871
MMA	43	0.741	0.934	50	0.649	0.757	26	0.961	0.530
Y-axis	42	0.381	0.913	18	0.428	0.272	11	0.888	0.224
FMA	27	0.964	0.586	60	0.714	0.909	44	0.897	0.897
SN-GoMn	41	0.914	0.891	50	0.694	0.757	32	0.914	0.653
LAFH/TAFH	11	0.222	0.239	39	0.649	0.590	8	0.961	0.163
R-angle	25	0.892	0.50	47	0.648	0.712	40	0.711	0.816

Positive predictive value; Sensitivity.

n = 161.

## DISCUSSION

In orthodontic diagnosis and treatment planning, it is essential to accurately assess an individual's facial skeletal pattern in all three dimensions, i.e. transverse, vertical and sagittal. The vertical facial pattern forms an important aspect in Orthodontics during the process of diagnosis and treatment planning by defining the variability in treatment planning, mechanics as well as in facial proportions.^11^ Tweed^20^ has related the stability of mandibular incisors after treatment based on vertical growth pattern. Since the vertical growth of face is the last to end, the assessment of facial discrepancy in the vertical dimension is not only important for an accurate diagnosis and an efficient treatment planning, it is of utmost significance to prevent relapse after the corrected malocclusion.

There are various skeletal parameters used to assess the vertical growth pattern of an individual. A multitude of times different parameters show conflicting results, and a specific diagnosis is hard to reach. Hence, this study focused on evaluating the diagnostic accuracy of various parameters so that the process of diagnosis may be limited to a minimal number of analyses. 

In the present study, a final diagnosis of vertical growth pattern was made on the basis of the results of the majority of analyses. The results of this final diagnosis were treated as the gold standard and were used to compare the diagnostic performance of the seven analyses using sensitivity, positive predictive value as well as Kappa statistics. The groups statistically matched well on the basis of age and sagittal patterns.

Correlation between various skeletal parameters has already been reported in the literature.^17^ ^,^ ^18^ ^,^ ^19^ In our study, all skeletal analyses showed a significant correlation with each other. A strong correlation was present between SN.GoGn and all the other skeletal analyses, except facial height ratio (LAFH.TAFH). MMA showed a moderate correlation with other skeletal analyses. Our results are in concordance with another study conducted by Asad and Naeem.^18^

SN.MP and SN.GoGn showed a strong correlation in both males (r = 0.898, *p* < 0.01) and females (r = 0.806). In contrast, Bahrou et al^17^ reported a moderate correlation between MMA and facial height ratio (LAFH.TAFH) in males (r = 0.550) and females (r = 0.497). The heterogeneity in results might be due to a difference in sample size. It is worth mentioning that any value of correlation does not relate to the diagnostic accuracy of any analysis.

Hence, in the present study, to compare the diagnostic agreement between skeletal analyses and the final diagnosis, Kappa statistics was applied. A substantial agreement was present between SN.GoGn and the final group (k = 0.850). Kappa statistics accounts whether a certain parameter indicates a specific vertical pattern simply by chance and provides more information than a simple correlation between two parameters.^21^

To evaluate validity of diagnostic indicators in identifying the vertical skeletal pattern, sensitivity was also calculated in each group separately. MMA showed a high sensitivity in the hypodivergent group (0.934), whereas FMA showed the highest sensitivity in normodivergent (0.909) and hyperdivergent groups (0.897). 

To further clarify whether a certain parameter can truly diagnose the vertical pattern, this study reports the positive predictive values (PPV) of all cephalometric parameters used in the present study. FMA yielded the highest PPV in the hypodivergent group (0.964), whereas SN.GoGn yielded the highest PPV in normodivergent (0.903) and hyperdivergent (0.897) groups. Thus, despite lesser sensitivity values, FMA and SN.GoGn proved to be more valid indicators in determining vertical skeletal pattern.

Multiple parameters can be used to evaluate the vertical growth pattern of an individual. In the present study, only the analyses commonly used during orthodontic diagnosis, indicating a specific growth pattern of the jaws in reference to the cranial base, were included. Other analyses, such as facial axis and facial depth angle, were excluded, as they indicate only chin position with respect to the cranial base.^22^ As new parameters are being proposed, analyses, such as R-angle, were also included in the study to check their reliability against commonly used analyses.^14^

With advances in digital imaging and tridimensional (3D) imaging technique, using the two-dimensional imaging technique (lateral cephalogram) to evaluate skeletal jaw relationship may be a potential limitation of this study.^23,24^ A survey of the current literature showed that although CBCT-generated images are better at evaluating skeletal jaw discrepancy, manual and digital lateral cephalograms are still reliable and valid for scientific research with the added advantage of a lower radiation dose.^25^ ^,^ ^26^ ^,^ ^27^

## CONCLUSIONS

1. A strong correlation was found between SN.GoGn and other skeletal vertical analyses, except facial height ratio (LAFH.TAFH).
2. SN.GoGn and FMA were found to be the most reliable indicators in assessing facial vertical growth pattern.
3. Facial height ratio (LAFH.TAFH) was found to be the least reliable indicator in assessing facial vertical growth pattern.

Hence, the number of cephalometric analyses to evaluate vertical skeletal jaw discrepancy may be reduced to a few analyses with higher diagnostic performance. This may result in accurate diagnosis and efficient treatment plan based on an individual's facial soft tissue pattern.
